# Clinical, epidemiological, and genotypic characteristics of rotavirus infection in hospitalized infants and young children in Yunnan Province

**DOI:** 10.1007/s00705-023-05849-9

**Published:** 2023-08-14

**Authors:** Hongjun Jiang, Yu Zhang, Xiaoyan Xu, Xiaohan Li, Yi Sun, Xin Fan, Ya Xu, Ting Su, Guiqian Zhang, Ziqin Dian

**Affiliations:** 1 Department of Clinical Laboratory, The First People’s Hospital of Yunnan Province, Kunming, 650100 Yunnan China; 2 Department of Pediatrics, The First People’s Hospital of Yunnan Province, Kunming, 650100 Yunnan China; 3 Department of Clinical Laboratory, Kunming Angel Women and Children’s Hospital, Kunming, 650032 Yunnan China

## Abstract

Rotaviruses are the most important pathogenic cause of non-bacterial diarrhea in infants and children. Approximately 60% of hospital admissions for acute diarrhea worldwide are caused by rotavirus infection. Rotavirus infection and hospitalization among children in China are a social burden, resulting in economic loss. The prevalence and geographical distribution of rotavirus genotypes is variable, partially due to population migration. Due to the unique geographical conditions and climate in Yunnan Province, several viruses with new genotypes have emerged, and multiple genotypes have become co-epidemic. In this study, rotavirus infection screening and genetic characterization of epidemic strains were performed in 149,492 infants and children admitted to hospitals in six representative prefectures in Yunnan Province between 2019 and 2021. The prevalence of rotavirus infection was 13.39% and was highest in January and lowest in September. G9P[8] was the main epidemic rotavirus genotype. Other epidemic genotypes included G2P[4], G8P[8], G9P[4], G2P[8], G3P[8], G4P[8], G3P[4], and G4P[6]. Phylogenetic analysis revealed that locally epidemic strains were influenced by importation of strains from neighboring provinces and other Asian countries. These findings provide a scientific basis for rotavirus prevention and control and lay a foundation for preliminary studies to establish a rotavirus surveillance network in Yunnan Province.

## Introduction

Rotaviruses are members of the family *Sedoreoviridae* that can infect humans and animals of all ages. They are most pathogenic in children under 5 years of age and in young animals and can cause severe acute diarrhea and death from dehydration, especially in areas with poor health care. Approximately 60% of hospital admissions of children with acute diarrhea worldwide are due to rotavirus infection. In China, the incidence of infection and rate of hospitalization of children due to rotavirus are 28-65% and 30-50%, respectively [1–3]. According to Centers for Disease Control survey data, the average cost of an outpatient visit for rotavirus diarrhea in children aged under 5 years in China is 168 RMB (approximately 23 USD), and the average hospitalization cost is 3145 RMB (approximately 440 USD). Thus, rotavirus diarrhea is a sizable social burden and cost. Rotavirus outbreaks have had a major impact on public health and socioeconomic development worldwide.

Rotaviruses are dsRNA viruses with a genome consisting of 11 segments of dsRNA of varying lengths, encoding a total of six structural and six non-structural proteins [4]. Rotaviruses are classified into eight recognized serotypes (RVA to RVH) and two pending serotypes (RVI, RVJ), according to the immunogenicity of the VP6 protein [5]. The surface protein, VP7, and the spike protein, VP4, are components of the outer capsid of the virion and are also important serum-specific antigens, which are used for serotype detection. Currently, serotype and genotype classification based on VP7 and VP4 is the most common typing method for rotaviruses. The genotype and serotype of VP7 are identified consistently, and both the genotype and serotype are represented as G. The VP4 serotype is more difficult to identify than its genotype, and P and P[n] are commonly used to represent the serotype and genotype, respectively. World Health Organization surveillance data show that G1 rotavirus is the most widespread in the world and is the dominant genotype in nearly all regions, followed by G9 and G8. G12 rotavirus is primarily distributed in the eastern Mediterranean region and Southeast Asia, and G3 rotavirus is more widely distributed in the Western Pacific region [6, 7].

Due to their genomic discontinuity, rotaviruses mutate and evolve through genetic rearrangements, recombination, and mutations rather than point mutations of single nucleotides [8]. When two genotypes of rotavirus carrying different gene fragments infect the same host at the same time, the newly synthesized gene fragments of each can randomly segregate and be packaged into the inner capsid protein of the virus, thus creating a new reassortant virus [9]. The emergence of reassortant viruses has greatly enriched the genetic diversity of rotaviruses.

The prevalence and geographic distribution of rotavirus genotypes are highly variable and are influenced by population migration. According to a 2014 report [10], typing results of 46,967 rotavirus cases from 81 countries between 2007 and 2012 showed that the most prevalent genotypes worldwide, in descending order, were G1P[8], G2P[4], G3P[8], G9P[8], G4P[8], and G12P[8]. Epidemiological surveillance in 10 regions of China between April 1998 and April 2000 found a total of 1305 rotavirus strains, and the most prevalent genotypes, in descending order, were G1 (72.7%), G2 (12.1%), G3 (14.2%), G4 (2.5%), and G9 (0.9%), with an additional 0.7% untyped and 3.1% mixed infections.

Yunnan Province in southwest China has 9.51 million children, borders several Southeast Asian countries, and is an important port of entry to China from South and Southeast Asia. Previous epidemiological studies have shown that the region is an important point of entry for viruses imported into China, and the genotype/subtype distribution of multiple viruses, such as HIV, hepatitis C virus, and dengue virus, is very complex. Therefore, this study was conducted to screen infants and children for severe rotavirus infection and perform genetic characterization of rotavirus strains in Yunnan Province to further understand the genetic diversity of rotaviruses and the genetic characteristics of major epidemics in the region, as well as to provide preliminary information for the development of a provincial rotavirus surveillance network.

## Materials and methods

### Selected population and sample collection

In this study, infants and children under 14 years of age who were admitted to hospitals in six prefectures of Yunnan Province (Kunming, Dali, Qujing, Dehong, Lincang, and Pu’er) between 2019 and 2021 were screened for rotavirus infection. This study was reviewed and approved by the Institutional Ethics Committee of the First People’s Hospital of Yunnan Province. The guardians of the children in this study were informed about the aims of the study and provided oral consent. Stool samples were collected within 1 h of excretion, and 1.5 g of sample was collected in a 1.5-mL centrifuge tube with an appropriate amount of isotonic phosphate-buffered saline containing a mixture of penicillin and streptomycin. The samples were mixed with the solution by trituration with a pipette and centrifuged at 8000 rpm for 30 min. All supernatants were aspirated into a new 1.5-mL centrifuge tube and stored in a -80°C freezer. Corresponding patient information was collected, including sex, age, and method of feeding. Clinical information was collected, including the presence of fever, vomiting, or an electrolyte imbalance and the severity of diarrhea.

### Virus antigen screening

Rotavirus antigen testing of fecal samples was performed using a Group A Rotavirus Test Kit (Wantai BioPharm, Beijing, China) according to the manufacturer’s instructions. The presence of two red bands, one in the test area and one in the quality control area, indicated that the sample was positive for rotavirus antigen.

### VP4 and VP7 gene amplification and phylogenetic analysis

RNA was extracted from 140 μL of fecal suspension using a TIANamp Virus RNA Kit (Tiangen Biotech Co., Ltd., Beijing, China), and the RNA quality was assessed using an ultraviolet spectrophotometer. The extracted RNA was aliquoted and stored at -80°C until use. The VP4 and VP7 genes were amplified by reverse transcription nested polymerase chain reaction (PCR) using methods and primers described previously [11]. PCR products were purified using an Agarose Gel DNA Extraction Kit (Takara, Dalian, China) and used for commercial sequencing (Sangon Biotech, Shanghai, China). All sequences of VP4 and VP7 genes of rotaviruses were submitted to the NCBI GenBank database under the accession numbers OR059471-OR060049 and OR060050-OR060627.

All sequences of VP7 and VP4 used in this study were aligned using the integrated Clustal X 1.83 program, and neighbor-joining trees were constructed in MEGA 6.0, using the Kimura 2-parameter model with gamma distribution and invariant sites as described previously. The reliability of the neighbor-joining trees was assessed by bootstrap analysis with 1000 pseudo-replicates. Values below 70% were excluded. The percentage of nucleotide sequence identity was calculated using an online tool (http://www.genome.jp/tools/clustalw/).

### Statistical analysis

Questionnaire data were entered using EpiData 3.0 software (The EpiData Association, Odense, Denmark). After error-checking, SPSS 20.0 software (IBM Corp, Armonk, NY, USA) was used for statistical analysis. Continuous data are expressed as the mean ± standard deviation and were analyzed using analysis of variance (ANOVA). Categorical data are expressed as the frequency and (%), and the χ^2^ test was used for comparisons between groups. Two-tailed *p*-values less than 0.05 were considered statistically significant.

## Results

### Characteristics of rotavirus infection in six prefectures of Yunnan Province

In this study, 149,492 infants and children were screened for rotavirus in six cities and towns in Yunnan Province during the study period. Of the patients, 47% were male and 53% were female, and the mean age was 27.69 months, with a range of 1-61 months. Antigen screening for rotavirus revealed a prevalence of 13.4% (20,013 cases). Among all children, 30,935 presented with acute or chronic diarrhea, and 56.0% (17,324 cases) of these were positive for rotavirus. Among children without diarrhea, 2.23% (2,689 cases) were positive for rotavirus.

**Fig. 1:**
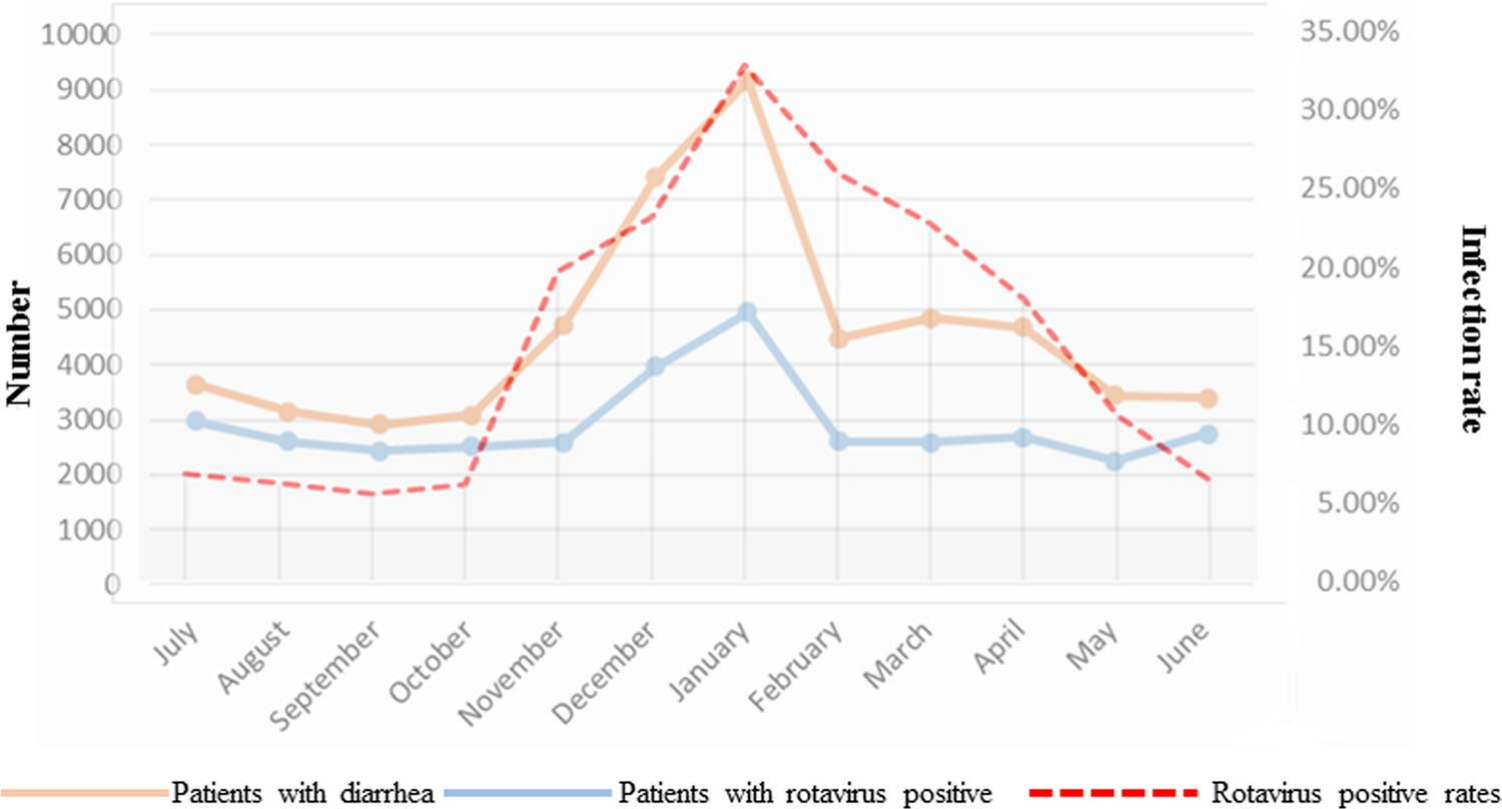
Prevalence of diarrhea, rotavirus infection, and proportion of patients testing positive for rotavirus by month

Monthly statistics showed that the number of admissions of neonates with clinically diagnosed diarrhea was highest in January. The prevalence of rotavirus infection was highest in January (4205/12804, 32.8%), followed by February (26.0%), December (23.2%), and March (22.7%), and was lowest in September (5.5%) (Fig. 1). As shown in Figure 1, the curves for the number of cases of diarrhea, the number of cases of rotavirus infection, and the prevalence of rotavirus infection had a similar trend. Most of the neonatal infections (12,408, 62.0%) occurred within the first week of life, with 4203 (21.0%) at age 1-4 weeks, 2956 (14.8%) at age 5-12 weeks, and 446 (2.2%) at > 12 weeks of age.

### Strain genotypes and clinical characteristics

**Fig. 2:**
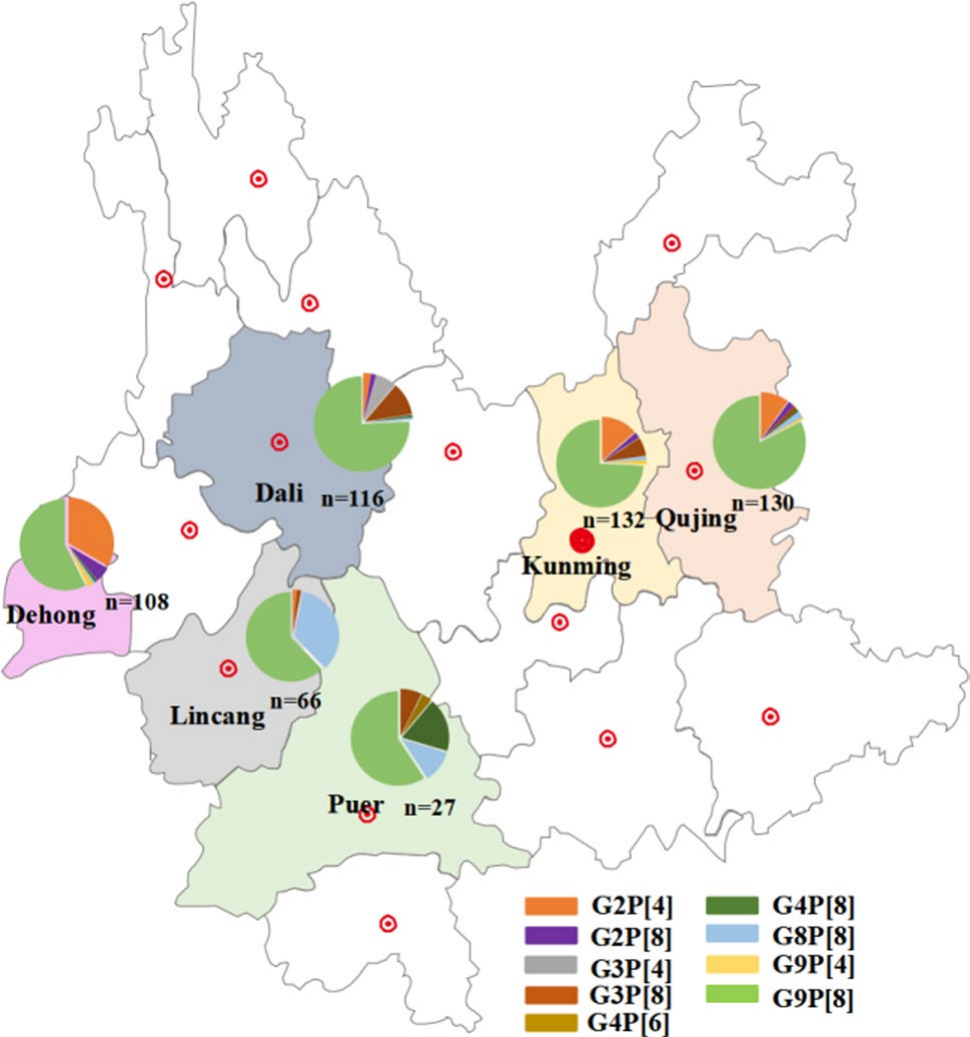
Rotavirus genotype distribution in six prefectures/cities in Yunnan

Among the 20,013 rotavirus-positive samples, a random sample of 3% (n = 604) of the positive samples was randomly selected to determine the genotype of the virus strains by phylogenetic analysis of their partial VP4 and VP7 genes. Of the 604 randomly selected samples, the VP4 and VP7 gene sequences were successfully obtained for 579 samples. Phylogenetic analysis showed that the rotavirus infections in infants and children in Yunnan Province included nine genotypes, among which G9P[8] (n = 412, 71.2%) predominated, followed by G2P[4] (n = 56, 9.7%), G8P[8] (n = 32, 5.5%), G9P[4] (n = 21, 3.6%), G2P[8] (n = 20, 3.5%), G3P[8] (n = 19, 3.3%), G4P[8] (n = 10, 1.7%), G3P[4] (n = 8, 1.4%), and one sample positive for G4P[6]. Distinct geographical distribution characteristics were observed through comparative genotype analysis of epidemic strains in different regions (Fig. 2) (*p* = 0.002). G9P[8] was the main epidemic strain in all prefectures/cities. However, the proportion of G8P[8] epidemic strains in Lincang and Pu’er was significantly higher than that in the surrounding areas. In addition to G9P[8], G3P[8] was also an important epidemic genotype in Dali (Fig. 2).

The clinical characteristics of the different genotypes, including fever, vomiting, electrolyte imbalance, diarrhea, and extraintestinal infections, were compared. The comparison revealed significant clinical differences in fever, vomiting, and diarrhea according to genotype. Significant differences were observed in the proportion of diarrhea caused by infection with different genotypes of viruses (*P *< 0.05), and G2P[4] was the genotype most likely to cause diarrhea. Children infected with G9P[8] and G8P[8] tended to have more-severe symptoms, whereas the incidence of fever and electrolyte imbalance did not differ significantly among the other genotypes (Table 1).

**Table 1:** Clinical characteristics of rotavirus genotypes

Genotype	Total	Clinical characteristics				
		Fever (Y/N)	Vomiting (Y/N)	Electrolyte disturbance (Y/N)	Diarrhea (Y/N)	Extraintestinal infection (Y/N)
G2P[4]	71	46/52	12/60	6/61	51/69	16/71
G2P[8]	14	8/10	2/8	0/4	9/12	1/7
G3P[4]	8	3/5	–	–	4/7	0/4
G3P[8]	27	20/25	7/23	3/22	17/27	3/20
G4P[6]	1	–	–	–	1/1	0/1
G4P[8]	8	3/8	2/8	1/7	5/8	0/6
G8P[8]	33	19/26	7/27	4/31	20/32	0/2
G9P[4]	5	2/5	1/4	0/4	3/5	0/5
G9P[8]	412	341/401	137/401	47/369	214/398	32/378
P value	–	0.0078	0.0156	0.0625	0.0039	0.1250

Y, the number of patients with fever, vomiting, electrolyte imbalance, diarrhea, and extraintestinal infections; N, the number of patients surveyed

### Phylogenetic characteristics of VP4 and VP7 genes of epidemic strains

**Fig. 3:**
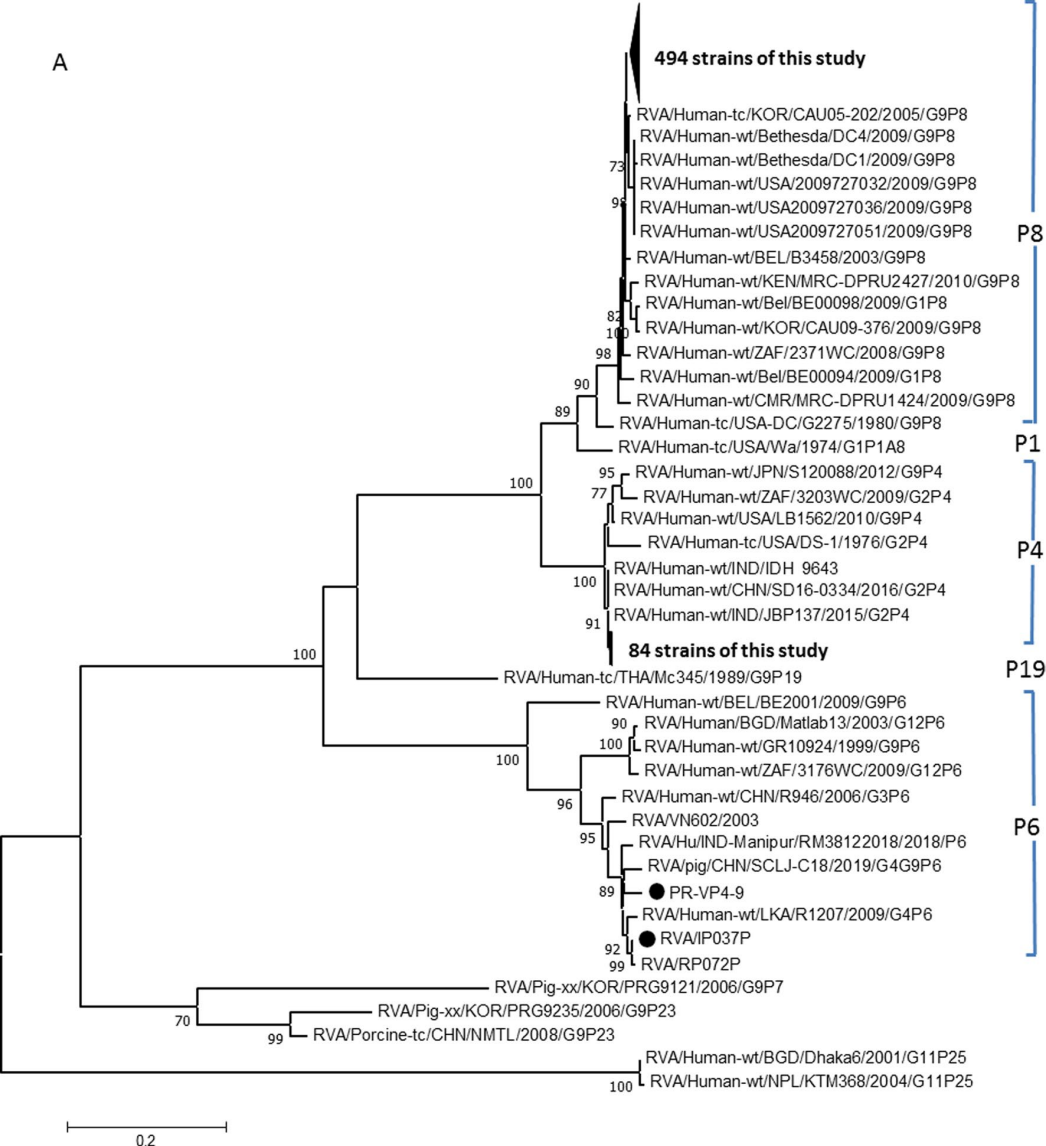

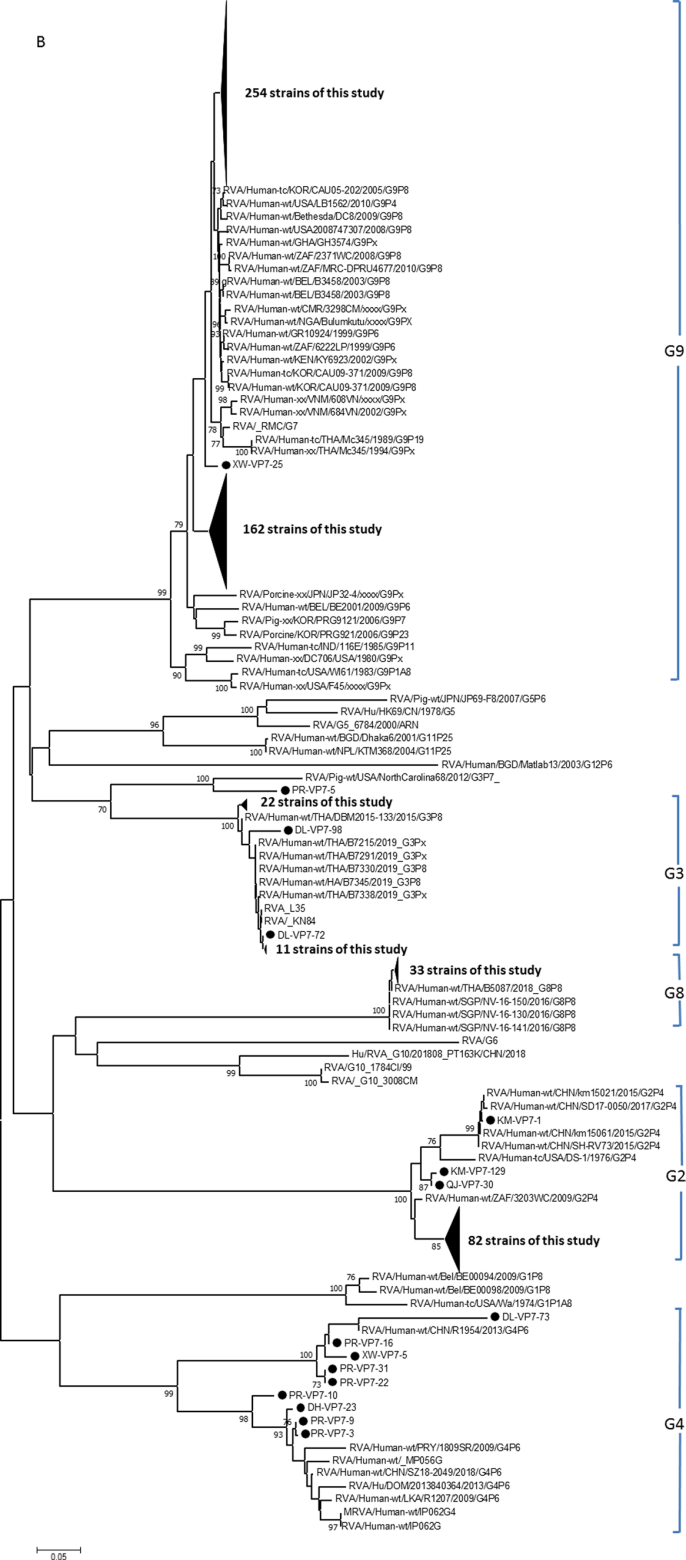
(a) Phylogenetic tree of rotavirus strains based on partial VP4 gene sequences. (b) Phylogenetic tree of rotavirus strains based on partial VP7 gene sequences. All samples from this study are indicated by dots

In order to further describe the genetic characteristics of the epidemic rotavirus strains in Yunnan Province, the 579 VP4 and VP7 gene sequences were analyzed by constructing phylogenetic trees with major epidemic virus strains in neighboring countries and regions and worldwide. The phylogenetic tree showed that the VP7 gene exhibited greater diversity than the VP4 gene. At least 12 branches were observed, and there was a large genetic distance between the different branches. The VP4 gene was relatively conserved, and most of the virus strains in this study had the P[8] genotype, whereas two strains had the P[6] genotype (Fig. 3a). The P[8] genotype strains in this study belonged to two different independent branches. The first lineage, which included 494 viral strains, showed closer genetic distance to strains from Japan and the United States, whereas the other lineage included viral strains that were more closely related to epidemic strains from India and Shandong Province in China.

A total of five VP7 genotypes were observed for the epidemic strains in this study (Fig. 3b), namely G2, G3, G4, G8, and G9. Among the strains in this study, G9 was the predominant genotype, followed by G2, G8, G3, and G4. The G9 epidemic strain exhibited a phylogenetic relationship very similar to that of P[8], with two independent branches, one very close to the strains from Korea, Japan, and Thailand, and the other showing a very high geographical aggregation, which appeared to be unique to Yunnan Province. The G2 epidemic strain was very closely related to strains reported in other provinces of China and in Kunming, the largest city in Yunnan Province, with some strains exhibiting 99.4-99.9% nucleotide sequence identity in the VP7 gene and a high degree of similarity to reference strains from Zhaotong Prefecture in Yunnan Province as well as neighboring countries, including Japan and Thailand. In addition, strains G3 and G4 showed similarity to strains from other parts of China and from other countries, whereas strain G8 showed similarity to strains from Thailand and Singapore in Southeast Asia. Notably, the closest relatives of G3 strain PRVP7-5 in this study were not strains infecting humans but instead strains infecting pigs in the United States, and these had a long genetic distance from strains of other origins.

## Discussion

In recent years, bacterial diarrhea has declined dramatically worldwide due to improved economic and health standards, but viral diarrhea caused by infection with intestinal viruses has become a major cause of acute gastroenteritis in infants and children, especially in developed and some developing countries. The primary viruses that cause acute gastroenteritis include rotaviruses, noroviruses, astroviruses, hepadnavirids, and adenoviruses. In this study, among the 579 rotavirus-positive cases, 32 were positive for norovirus, with one case of genotype I, 31 cases of genotype II, three cases of genotype IV, and two cases of mixed genotype II and IV infections (data not shown).

Rotaviruses remain the primary pathogens causing diarrhea in infants and children. Nearly 40,000 children die each year in China due to diarrhea caused by rotaviruses, accounting for about 12% of all deaths in children under 5 years of age in China. Outbreaks of rotavirus have been documented in various provinces and cities in China [12–14]. In this study, the prevalence of rotavirus positivity among infants and children admitted to hospitals in six prefectures/cities of Yunnan Province was 13.39%, and 56.00% among patients with diarrhea, which is similar to that previously reported in Kunming [15]. Comparative analysis of the prevalence of infection in different age groups showed that rotavirus infection was detected in children of all ages, with the prevalence of infection gradually decreasing with increasing age. In addition, there was a correlation between age and the seasonal prevalence, consistent with other reports [11, 12]. Although the incidence of rotavirus infection varies by country and region, transmission is generally higher in the autumn and winter. Kunming has high temperatures between April and September. The low prevalence of rotavirus positivity and the high prevalence of acute gastroenteritis in children during these months is probably primarily due to diarrhea caused by bacteria. In addition, some other intestinal viruses, including noroviruses, astroviruses, and adenoviruses, can cause diarrhea, and the high prevalence of these pathogens makes the rotavirus prevalence lower than that of acute gastroenteritis.

Genotypic analysis of the local epidemic strains showed that G9P[8] is the predominant genotype strain in Yunnan Province. G1P[8], which was once epidemic worldwide, was not detected in this study, and G3P[8] was rare, suggesting that these two genotypes are no longer epidemic and that they have been replaced by new genotypes. In this study, the epidemic rotavirus genotypes differed in different prefectures of Yunnan Province: the G2P[4] genotype was sub-epidemic in western Yunnan, including Dehong Prefecture, whereas in Pu’er and Lincang, which are more southern, the epidemic G8P[8] genotype was significantly more prevalent. This may be related to the nomadic lifestyle of the populations in these areas.

In this study, the G9 sequences in the Kunming region showed a high degree of similarity to sequences from East Asian countries, such as Korea and Japan, as well as other parts of China, suggesting that this virus can spread to some extent among neighboring countries and regions, with some strains becoming regionally dominant. By 1983, after its first discovery in the United States [16], the prevalence of G9 rotavirus increased in some regions of the world, with the G9P[8] genotype also being the earliest epidemic genotype. In China, the G9 virus was first discovered in Yunnan in 1998 [17] and thereafter was only reported sporadically as a rare genotype until 2011, when G9P[8] rapidly replaced G1P[8] and G3P[8] as the dominant genotype in Nanjing and Wuhan. G9P[8] rotaviruses subsequently showed explosive growth in China in a short period of time, and the following two years of surveillance showed that this genotype dominated the epidemic in many provinces and cities across China. Similar to Nanjing and Wuhan, G1P[8] and G3P[8] were the dominant genotypes in Kunming before 2006, but due to a lack of epidemiological surveillance, the epidemiological changes of local rotavirus genotypes and the molecular epidemiological correlates in the subsequent decade are unknown. The current survey showed that the G9P[8] genotype has become the dominant epidemic genotype in Yunnan Province, as it is in other provinces, and it is possible that this genotype has become the dominant genotype in China and Southeast Asia through cross-regional and cross-country transmission.

The G3 strain PRVP7-5 from this study showed the closest relationship to a virus strain reported to infect pigs in the United States that exhibited a long genetic distance from strains from other sources, suggesting acquisition of gene fragments of animal origin by reassortment. Rotaviruses have a wide host range, and in addition to humans, mammals such as swine, cattle, cats, and dogs and also birds such as swans, pigeons, and turkeys can be infected with rotaviruses. Therefore, when two genotypes of rotavirus carrying different gene fragments infect the same host at the same time, the newly synthesized gene fragments of each can randomly segregate and be packaged into the inner capsid protein of the virus, thus creating a new reassortant virus [9]. The emergence of reassortant viruses has greatly enriched the genetic diversity of rotaviruses. This phenomenon is especially common in G9 rotaviruses, which have gradually become more prevalent in recent years and most often infect pigs. By 2011, at least 40 of the more than 50 rotavirus genotypes that have been identified had been found in humans [18], and multiple-reassortant viruses containing fragments of animal rotavirus genes have been identified in affected children. Notably, these reassortant viruses are usually discovered because they cause severe diarrhea [19]. The exact mechanism by which these animal rotaviruses infect humans and reassort with human rotavirus strains needs to be investigated in depth.

This study has some limitations. Although the genotyped samples were selected randomly and should be representative of all samples, the possibility of selection bias cannot be ruled out. Different clinical characteristics have been reported with infections by different genotypes [14, 16]. Studies with a larger sample size are needed to confirm the observed differences in clinical characteristics between genotypes. In this study, the small number of some genotypes may have introduced some bias to the analysis.

In conclusion, infants and children aged 0-5 years admitted to hospitals in selected areas of Yunnan Province screening for rotaviruses had a similar prevalence of infection to those in other localities in China. The predominant local epidemic genotype was G9P[8]. Most of the genotypes identified in this study were closely related to genotypes from other provinces in China and are likely to be of Asian or Southeast Asian origin and to have spread locally through population migration, similar to other bloodborne viruses in Yunnan Province [20–23]. This suggests a need for enhanced surveillance of cross-border virus importation in Yunnan Province.

## Data Availability

The data are available from the corresponding authors on reasonable request.

## References

[1] Orenstein EW, Fang ZY, Xu J (2007). The epidemiology and burden of rotavirus in China: a review of the literature from 1983 to 2005. Vaccine.

[2] Ramig RF (2004). Pathogenesis of intestinal and systemic rotavirus infection. J Virol.

[3] Julian TR (2016). Environmental transmission of diarrheal pathogens in low and middle income countries. Environ Sci Process Impacts.

[4] Donelli G, Superti F (1994). The rotavirus genus. Comp Immunol Microbiol Infect Dis.

[5] Steele JC (1999). Rotavirus. Clin Lab Med.

[6] Matthijnssens J, Bilcke J, Ciarlet M (2009). Rotavirus disease and vaccination: impact on genotype diversity. Future Microbiol.

[7] Esona MD, Ward ML, Wikswo ME (2021). Rotavirus genotype trends and gastrointestinal pathogen detection in the United States, 2014–2016: results from the new vaccine surveillance network. J Infect Dis.

[8] McDonald SM, Aguayo D, Gonzalez-Nilo FD, Patton JT (2009). Shared and group-specific features of the rotavirus RNA polymerase reveal potential determinants of gene reassortment restriction. J Virol.

[9] Bányai K, Mijatovic-Rustempasic S, Hull JJ (2011). Sequencing and phylogenetic analysis of the coding region of six common rotavirus strains: evidence for intragenogroup reassortment among co-circulating G1P[8] and G2P[4] strains from the United States. J Med Virol.

[10] Desselberger U (2014). Rotaviruses. Virus Res.

[11] Dian ZQ, Fan M, Wang BH (2017). The prevalence and genotype distribution of rotavirus A infection among children with acute gastroenteritis in Kunming, China. Arch Virol.

[12] Fang ZY, Wang B, Kilgore PE (2005). Sentinel hospital surveillance for rotavirus diarrhea in the People's Republic of China, August 2001–July 2003. J Infect Dis.

[13] Wang YH, Pang BB, Ghosh S (2014). Molecular epidemiology and genetic evolution of the whole genome of G3P[8] human rotavirus in Wuhan, China, from 2000 through 2013. PLoS One.

[14] Wang XY, Xu ZY, von Seidlein L (2005). Incidence of diarrhea caused by rotavirus infections in rural Zhengding, China: prospective, population-based surveillance. J Infect Dis.

[15] Kang Y, Cai Y (2018). Epidemiology and genetic diversity of rotavirus in Kunming, China, in 2015. Intervirology.

[16] Clark HF, Hoshino Y, Bell LM (1987). Rotavirus isolate WI61 representing a presumptive new human serotype. J Clin Microbiol.

[17] Li Y, Wang SM, Zhen SS (2014). Diversity of rotavirus strains causing diarrhea in <5 years old Chinese children: a systematic review. PLoS One.

[18] Tsugawa T, Hoshino Y (2008). Whole genome sequence and phylogenetic analyses reveal human rotavirus G3P[3] strains Ro1845 and HCR3A are examples of direct virion transmission of canine/feline rotaviruses to humans. Virology.

[19] Dunn SJ, Greenberg HB, Ward RL (1993). Serotypic and genotypic characterization of human serotype 10 rotaviruses from asymptomatic neonates. J Clin Microbiol.

[20] Zhang Y, Vrancken B, Feng Y (2017). Cross-border spread, lineage displacement and evolutionary rate estimation of rabies virus in Yunnan Province, China. Virol J.

[21] Wang B, Liang Y, Feng Y (2015). Prevalence of human immunodeficiency virus 1 infection in the last decade among entry travelers in Yunnan Province, China. BMC Public Health.

[22] Wang B, Feng Y, Li Z (2014). Distribution and diversity of hepatitis B virus genotypes in Yunnan, China. J Med Virol.

[23] Wang B, Liang Y, Yang S (2018). Co-circulation of 4 dengue virus serotypes among travelers entering China from Myanmar, 2017. Emerg Infect Dis.

